# Modulable 3D-printed plantibody-laden platform enabling microscale affinity extraction and ratiometric front-face fluorescence detection of microcystin-LR in marine waters

**DOI:** 10.1007/s00604-024-06547-2

**Published:** 2024-07-27

**Authors:** Roser Payà-Pou, Julia Aguirre-Camacho, Ernesto Francisco Simó-Alfonso, Dietmar Knopp, Manuel Miró, Enrique Javier Carrasco-Correa

**Affiliations:** 1 CLECEM Group, Department of Analytical Chemistry, University of Valencia, C/ Doctor Moliner, 50, 46100 Burjassot, Valencia Spain; 2 Department of Chemistry, Chair of Analytical Chemistry and Water Chemistry, Technical University Munich, TUM School of Natural Sciences, Lichtenbergstrasse 4, 85748 Garching, Germany; 3 FI-TRACE Group, Department of Chemistry, University of the Balearic Islands, Carretera de Valldemossa, km 7.5, Palma, 07122 Spain

**Keywords:** 3D printing, Emerging contaminants, Immunoassay, Seawater

## Abstract

A 3D-printed stereolithographic platform for selective biorecognition is designed to enable convective microscale affinity extraction of microcystin-LR (MC-LR) followed by direct solid-phase optosensing exploiting ratiometric front-face fluorescence spectroscopy. For this purpose, a recombinant monoclonal plantibody (recAb) is covalently attached to a 3D-printed structure for sorptive immunoextraction, whereupon the free and unbound primary amino moieties of the recAb are derivatized with a fluorescent probe. The fluorophore-recAb-MC-LR laden device is then accommodated in the cuvette holder of a conventional fluorometer without any instrumental modification for the recording of the solid-phase fluorescence emission. Using Rodbard’s four-parameter sigmoidal function, the 3D-printed bioselective platform features a limit of detection (LOD) of 28 ng L^−1^ using a sample volume of 500 mL, device-to-device reproducibility down to 12%, and relative recoveries ranging from 91 to 100% in marine waters. Printed prototypes are affordable, just 0.4 € per print and ≤ 10 € per device containing recAb. One of the main assets of the miniaturized immunoextraction device is that it performs comparably well in terms of analytical figures of merit with costly mass spectrometric-based analytical methodologies, such as HPLC–MS/MS. The device is readily applicable to high-matrix samples, such as seawater, as opposed to previous biosensing platforms, just applied to freshwater systems.

**Graphical abstract:**

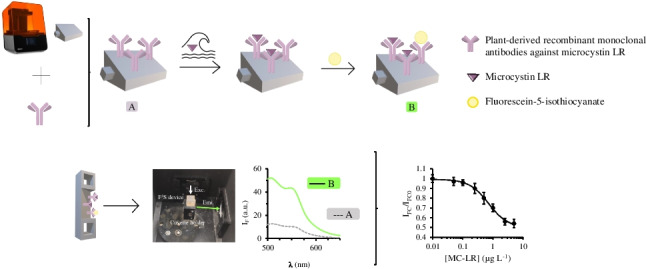

**Supplementary Information:**

The online version contains supplementary material available at 10.1007/s00604-024-06547-2.

## Introduction

Microcystin-LR (MC-LR), a hepatotoxic cyanotoxin released by the cyanobacteria so-called blue-green algae, is an emerging environmental pollutant that poses severe risks to both aquatic ecosystems and human health [1]. The prevalence of extensive algal blooms in coastal habitats due to eutrophication and climate change is principally linked to the occurrence of MC-LR in seawater [2]. MC-LR is a potent and specific inhibitor of phosphatases, which might lead to cytoskeleton disruption and DNA damage in both mammals and higher plants [3]. In fact, chronic exposure to MC-LR and class analogs thereof might challenge the human digestive, respiratory, reproductive, circulatory, and nervous systems/environments [4]. Hence, the World Health Organization (WHO) suggested a provisional 1 µg L^–1^ MC-LR guideline as the maximum allowed concentration in drinking water [5] and the Directive (EU) 2020/2184 on the quality of water intended for human consumption corroborated such concentration as a parametric value [6]. Indeed, MC-LR and its analogs are deemed contaminants of emerging concern and thus there is a quest to monitor their occurrence in environmental waters. In fact, the concentrations of microcystins in fresh, estuarine, and marine waters vary drastically from a few to hundreds or thousands of µg/L depending on the magnitude and length of the algal bloom [7].

To identify and determine microcystin analogs in environmental waters, high-performance liquid chromatography (HPLC) is commonly coupled with an ultra-violet/visible (UV/vis) or mass spectrometry (MS) detection [8]. In this sense, HPLC–UV/vis offers routine methods while HPLC–MS enables enhanced sensitivity and selectivity. Nevertheless, the former is unable to differentiate structural variants of microcystins, and the latter might be prone to severe matrix effects while necessitating bulk and expensive instrumentation, and skilled personnel for operation. Protein and DNA-incorporated bioselective platforms including enzyme-linked immunosorbent assays (ELISA) [9], immunosensors [10, 11], and aptasensors [12] are appealing alternatives to their chromatographic counterparts. In fact, biosensors with immobilized antibodies (Abs) or aptamers, are highly selective and easy to handle, offer decentralized detection, and are amenable to a plethora of transduction systems for enhanced detectability [13]. However, there are two main limitations: (i) method development and optimization of functional biosensing prototypes using standard manufacturing techniques might be time and cost-consuming and (ii) the use of animal models in bioassays (including preparation of murine monoclonal Abs) is not recommended by the European REACH legislation [14].

3D printing has recently emerged as a promising additive manufacturing technology to cope with the need for specialized, customizable, and cost-effective optical and electrochemical sensing devices [15]. 3D printing is a layer-by-layer fabrication technique that enables the designing of bespoke 3D objects. Low force stereolithography (LFS), as a subtype of stereolithography (SLA) [15, 16], is deemed particularly intriguing for prototyping affordable hardware components, scaffolds, housing, and fully integrated devices in the field of (bio)analytical chemistry [17]. A dedicated laser is leveraged in LFS to polymerize a (meth)acrylate-laden liquid resin that enables the construction of point-by-point and layer-by-layer functional objects. The 3D-printed LFS-based devices have found numerous applications in the analytical chemical field for sample preparation and separation using tailorable sorptive phases [16, 18, 19], components of detection systems [20], and functional microfluidic platforms [21]. However, the opportunities of LFS for the production of sensor devices have not been fully explored as of yet [15]. In fact, 3D printing offers itself as an invaluable springboard for developing customizable and unrivaled functional and multi-purpose (bio)sensing platforms that enable integrating innovative extraction/sensing functionalities at will [17]. In this context, the ability to create novel devices with Lego-like modular connections presents an exciting opportunity for 3D printing. This approach enables the individual preparation of distinct functional components, which can be subsequently assembled to build a customized, fit-for-purpose 3D-printed device.

This work gears toward the fabrication of a 3D-printed modulable Lego-type platform with a bioselective surface obtained through the covalent attachment of antibodies. The fabricated device is aimed at accommodating microscale affinity extraction and detection by ratiometric front-face fluorescence spectroscopy (F^3^S) without the need for analyte elution [22, 23]. To the best of our knowledge, plant-derived recombinant monoclonal antibodies (recAb) are for the first time used in combination with 3D printed structures. In fact, the production of recombinant proteins in plants offers the necessary economy and scalability to obtain low-cost antibodies with great potential for use in biosensors and purification matrices [24]. LFS is herein harnessed to design customized 3D printed open-source solid devices that reproduce the dimensions of the cuvettes of conventional bench-top fluorescence spectrometers and thus they are prototyped to fit commercial equipment. The applicability of the immunoextraction device is demonstrated by the trace level determination of MC-LR selectively in troublesome samples, such as saline waters.

## Experimental

Detailed information of (i) reagents and materials, (ii) instrumentation, (iii) preparation of plant-derived recombinant antibody against MC-LR, and (iv) 3D printed surface modification (including a reaction scheme, Figure S1) is available as Supplementary Information (SI). The design and fabrication of the 3D printing device and the immunoextraction procedure are described below.

### Design and 3D printing of the modulable platforms

**Fig. 1:**
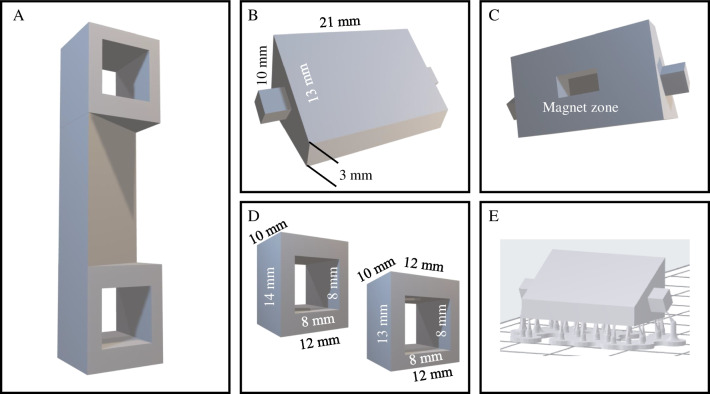
Illustration of the assembled 3DF^3^S functional unit (**A**) and its components: the central recAb-laden module (frontal face, **B** and back face showing the cavity for magnet, **C**) and the lateral supports (**D**). The main unit with supports as oriented onto the moving LFS printer platform is shown in **E**

The 3D models were designed using the freeware FreeCAD® (Fig. 1). Prototypes were modeled for bearing the F^3^S angles required to measure fluorescence on solid surfaces [23]. In this particular case, the device (Fig. 1A) consisted of three elements: the central detection module, the surface of which was decorated with recAb (Fig. 1B), with the back face containing a cavity for a small magnet to be used in solid-phase microextraction protocols (Fig. 1C); and two lateral supports that enable positioning of the detection platform inside the cuvette holder of the spectrofluorometer (Fig. 1D). The solid phase optosensing platform was built as a Lego system with plug-and-play exchangeable modules.

The computer-aided design (CAD) devices were printed horizontally (see Fig. 1E) using the printer’s adjustable layer height with the addition of 3 mm supports to facilitate their subsequent removal from the printing platform. In any case, no support was added to the surface to be further modified by anchoring the recAb (Fig. 1E). The green-state 3D F^3^S print was then subjected to a post-processing procedure to remove any remnants of resin and oligomers while enabling the completion of the radical polymerization reaction. For this purpose, the printed devices were subsequently immersed in isopropyl alcohol (IPA), water, and IPA again for 15 min each in an ultrasonic bath. Then, the 3DF^3^S units were dried under nitrogen stream and placed in the UV light chamber for 1 h for post-curing.

### Immunoaffinity extraction and F^3^S detection protocols

First, the fluorescence emission spectrum of the recAb-immobilized 3D printed module was obtained before initiating the immunoextraction procedure at λ_exc_ = 492 nm. The full emission spectrum was used as a reference for further measurements of standards and samples. Subsequently, MC-LR was extracted from standards or samples (ranging from 50 to 500 mL) in 10 mM phosphate-buffered saline (PBS) at pH 7.4. For this purpose, a 3D-printed immunodevice enclosing a mini-magnet (see Fig. 1C) was placed in a beaker and agitated at 300 rpm for 30 min at 35 °C. The experimental conditions of the sorptive microextraction protocol were adopted from previous affinity studies with exactly the same recAb [25], and our own experience on immunoaffinity extraction exploiting 3D printing devices [18]. After the extraction process, the unit was thoroughly cleaned with 10 mM PBS and dried using a gentle stream of N_2_. Then, the front face of the 3D printed structure was exposed to 100 μL of a fluorescein-5-isothiocyanate (FITC) solution (15 mmol FITC per 1 mmol of recAb) for 30 min at room temperature. Finally, the unit was dried once again with N_2_, and the fluorescence emission spectrum was recorded again. The ratiometric fluorescence emission signal (*I*_FC_) was obtained at an excitation wavelength of 492 nm using the following equation:

1 $${I}_{FC}=\frac{{I}_{517a}-{I}_{517b}}{{I}_{626a}-{I}_{626b}}$$

in which *I*_*517a*_ and *I*_*626a*_ are the fluorescence emission signals obtained at 517 and 626 nm, respectively, after MC-LR extraction and recAb derivatization, and *I*_*517b*_ and *I*_*626b*_ are the fluorescence emission signals at 517 and 626 nm, respectively, of the 3D print before starting the extraction protocol. Indirect detection of the target is, in this work, enabled because of the derivatization of the free amino moieties of the antibody, after MC-LR-recAb interaction, with FITC so that the larger the concentration of the analyte the lower the analytical response.

## Results and discussion

### Prototyping of the 3D-printed immunoaffinity scaffold for F^3^S detection

**Fig. 2:**
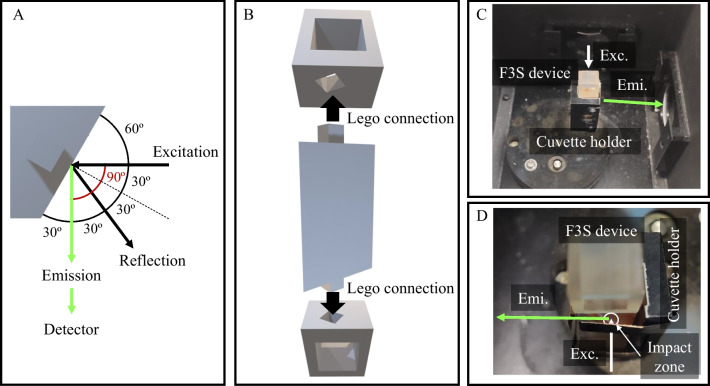
Excitation, reflection, and emission angles on the 3D-printed main module of the 3DF^3^S device (**A**). Lego-like connections between the main unit and the supports in upright orientation as introduced in the spectrofluorometer (**B**). Pictures of the 3D-printed design after placement in the cuvette holder of the spectrofluorometer (**C** and **D** as magnified view)

The design of the principal module of the 3D printed scaffold was conducted by thorough consideration of the distinct angles required for the successful execution of F^3^S, as previously described by Riza et al. [22]. Figure 2A outlines the angles of excitation, reflection, and emission on the main unit of the 3DF^3^S device (see Fig. 1A) once placed in the holder of the spectrofluorometer.

As depicted in Fig. 2A, the unique geometry and orientation of the print enables the acquisition of the emission light at 90º from the excitation beam (Fig. 2A, red angle). Moreover, the 3D scaffold prototype ensures that light reflection occurs at 60º from the excitation beam, effectively minimizing light scattering effects (Fig. 2B, black angles) [23]. Although the 3D-printed device encompasses all the necessary components for F^3^S detection (Fig. 1A), three separate units were printed out: (i) the central module (Fig. 1B), and (ii and iii) the two lateral supports (Fig. 1D). Initially, we aimed at a one-step printed unit. Because of the cubic nature of the lateral supports, however, covalent functionalization of just the front face of the central module became challenging. Therefore, a Lego-like platform was designed instead for successful modification of the detection unit only while facilitating the subsequent attachment to the lateral supports before the assembled solid 3DF^3^S device could be used in standard instrumentation (Fig. 2B). For this purpose, the 3D-printed central unit and supports were equipped with appropriate press-fit connectors (Fig. 2B) to ensure seamless integration. Furthermore, the lateral supports were thoughtfully designed as open cubes, thus effectively minimizing the unnecessary use of resin while maintaining their structural integrity.

The investigation of the various chemical derivatization reactions for covalent attachment of the Abs onto the surface of the LFS print was conducted previously in our lab [18]. In any case, the 3D printed surface was herein examined by scanning electron microscopy (SEM), as illustrated in Figures S2A and S2B. Figure S2A displays the surface of the non-modified surface of the 3DF^3^S device and evinces printing layers distant from 150 µm each other. Conversely, after undergoing covalent decoration, including recAb attachment (Figure S2B), a rougher surface is observed, yet the underlying printing layers remain still visible. To thoroughly characterize the anchorage of the recAb onto the 3DF^3^S device, X-ray energy dispersive analysis (EDAX) was used for indirect detection of the Ab based on the content of S-containing amino acids [26]. Figure S2C shows the S to C percentage ratio for both the unmodified and modified scaffold with increasing concentrations of recAb from 0.1 to 1 mg mL^–1^. The experimental data corroborated the successful attachment of the recAb, as demonstrated by the direct linear relationship (*R*^2^ = 0.996) between the percentage of S against the recAb concentration in the reaction mixture. Additionally, the nitrogen content of the 3D printed platform was quantitatively assessed through elemental analysis before and after the reaction with the recAb. The total number of nitrogen atoms accounting for both the light and heavy chains of the recAb is 1676 per antibody molecule (MW = 150 kDa). Based on the elemental analysis of N, the reaction yield was determined to be 58%. Consequently, the amount of recAb immobilized onto the 3D-printed surfaces ranged from 2.1 to 21.2 µg cm^–2^, corresponding to nominal recAb concentrations of 0.1–1 mg mL^–1^.

### Investigation of the critical variables for F^3^S ratiometric detection following immunoaffinity extraction

**Fig. 3:**
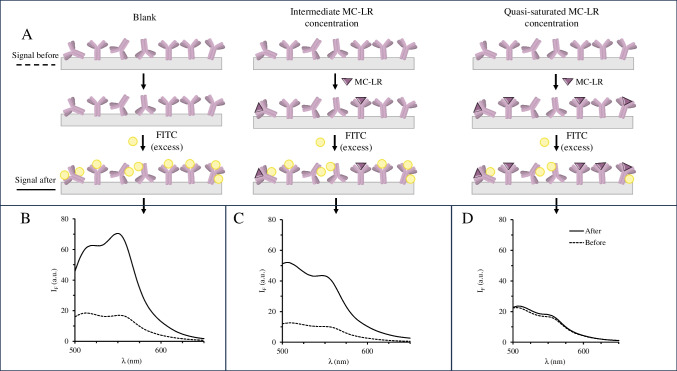
Proposed detection mechanism of the 3DF^3^S device based on the available amino groups at the CDRs of the antibody after the immunoassay, and further chemical reaction with FITC (**A**). The figure shows 3 different scenarios: absence of MC-LR or blank (left hand), intermediate MC-LR concentration (center), and high MC-LR concentration (right hand). **B**–**D** shows the experimental emission spectra at a 492-nm excitation wavelength after (–) and before (--) the immunoextraction of MC-LR and derivatization with FITC (2.4 mg L^–1^) at various concentrations of MC-LR: 0 (**B**), 0.25 (**C**), and 2.5 (**D**) µg·L^–1^, equating to blank, intermediate, and elevated concentrations in panel A. The 3DF^3^S devices were modified, in all cases, with 0.5 mg mL^–1^ of recAb in 10 mM PBS prior to use. Note: The differences observed across the fluorescence spectra of the Ab-laden devices (“Signal Before”) illustrate the variability of the prints and recAb covalent immobilization method

The selection of the excitation/emission fluorescence wavelengths for the FITC derivatization reaction was initially based on those recommended by the manufacturer [27], i.e., 492 and 517 nm for the excitation and emission wavelengths, respectively. Preliminary tests were performed to investigate the non-specific reactivity of FITC against the primary amino moieties of the 3D-printed immunoaffinity device after extraction of MC-LR within the concentration range of 0–2.5 µg L^–1^. Figure 3 shows the emission spectra of three different recAb-containing 3DF^3^S platforms before immunoextraction and without FITC (Fig. 3, dashed lines), and after immunoextraction with MC-LR and chemical reaction with FITC (Fig. 3, continuous lines) at three concentration levels of MC-LR, viz*.,* 0, 0.25, and 2.5 µg L^–1^. As it can be observed, the recAb-loaded 3DF^3^S device bears inherent fluorescence emission but this is enhanced after chemical derivatization with FITC with two characteristic bands (517 and 553 nm). The 517 nm band is characteristic of the FITC probe, yet that at 553 nm is not expected in derivatization reactions in solution. Therefore, it is attributed in our case to the combination of the distinct energy levels of FITC, recAb, and the polymeric structure of the 3D-printed device. In any case, a poor linear correlation of the MC-LR concentration against fluorescence emission at 553 nm was found. An interesting observation is the behavior of the fluorescence emission intensity after MC-LR immunoextraction at 517 nm. The fluorescence intensity does not increase with increasing concentrations of MC-LR despite bearing primary amines. This observation, as shown in Fig. 3A, can be explained by the covalent bonds that FITC generates with available unbound amino moieties of the recAbs that are attached to the surface of the 3DF^3^S device. However, after MC-LR recognition, the side chain amino groups from glutamine, lysine, and arginine of the light and heavy chains of the complementary determining regions (CDRs) of the rec-Ab [25], do not undergo a chemical reaction with FITC. In other words, the amino moieties at the CDRs of the recAb paratope that recognizes the antigen, as demonstrated by docking studies elsewhere [28], are after loading with MC-LR no longer available for chemical reaction with FITC. With high concentrations of MC-LR, the majority of paratopes are bound to targets. Thus, recAbs are less available to react covalently with FITC, leading to either a slight increase or no change in fluorescence emission intensity with respect to that before the immunoassay and chemical derivatization with FITC (Fig. 3A right hand and Fig. 3D). Conversely, at intermediate concentrations of MC-LR (Fig. 3A center and Fig. 3C), the fluorescence emission intensity after MC-LR binding is higher than that observed before immunoextraction and FITC reaction. However, in all cases, it is lower than obtained in the absence of an analyte (blank signal) because most of the amino moieties of the recAb are free for non-specific chemical derivatization with the fluorotag (Fig. 3A, left hand and Fig. 3B).

Notwithstanding the experimental data depicted in Fig. 3, the analytical signals -obtained at either 517 nm or 553 nm after immunoextraction and FITC derivatization, and subtraction of those of the inherent fluorescence of the 3D prints- did not show any direct statistical correlation with MC-LR concentrations. To tackle this issue, Eq. (1) (See Experimental) was proposed for ratiometric fluorescence intensity (I_FC_) detection. To this end, a ratio of the fluorescence emission at 517 nm against that of a wavelength for which neither the 3DF^3^S device nor the FITC yielded significant emission, viz., 626 nm, was used for baseline correction. The fluorescence emission in the absence of analyte was normalized to 1 so as to obtain the typical log X-axis sigmoidal calibration graphs based on the symmetrical (four-parameter) logistic dose–response curves (Eq. 2) used for immunoaffinity extraction systems [29], as shown in Figure S3.

2 $$\frac{{I}_{FC}}{{I}_{FC0}}=d+\frac{a-d}{1+{\left(\frac{[MC-LR]}{c}\right)}^{b}}$$

in which *I*_*FC*_ is the ratiometric fluorescence emission obtained by Eq. (1), *I*_*FC0*_ is the ratiometric fluorescence emission signal at [MC-LR] = 0 µg L^−1^, *a* is the theoretical response at zero concentration, *b* is the slope factor, *c* is the mid-range concentration (inflection point), *d* is the theoretical response at infinite concentration, and [MC-LR] is the analyte concentration in 50 mL-standard volume.

Akin to competitive immunoassays, the concentration of recAb for the fabrication of the 3D printed immunoextraction scaffold was evaluated within the range of 0.1–1.0 mg mL^–1^. The dynamic range is jeopardized at lower concentrations on account of insufficient Ab while the surplus above 1.0 mg mL^–1^ compromises the sensitivity of the method for MC-LR. Under the above experimental conditions, the analytical figures of merit of the resulting Rodbard’s logistic sigmoidal curves, namely, LOD, the limit of quantification (LOQ), device-to-device precision (given as relative standard deviation, RSD%, *n* = 3), and linear range, were examined. Table S1 lists the analytical parameters examined for the four sigmoid curves displayed in Figure S3, including the linear ranges for I_FC_/I_FC0_ *vs.* log [MC-LR]. LOD and LOQ (*n* = 5) were calculated for a 50-mL blank solution as the concentrations equating to 1 − 3s_b_ (standard deviation of blank) and 1 − 10s_b_, respectively, using the ratiometric calibration graphs. 3DF^3^S platforms fabricated with 0.1 mg mL^–1^ recAbs exhibited the best LOD and LOQ values, but the inter-device precision and the linear range were poorer compared to the 3DF^3^S platforms prepared with 0.25 mg mL^–1^ or 0.5 mg mL^–1^ recAb. On the other hand, the 3DF^3^S devices prepared with 1.0 mg mL^–1^ showed the highest LOQ value, which does not suffice for environmental assays. This is due to the recAb surplus as expected from indirect measurements in competitive reactions. Therefore, the 3DF^3^S devices that showed the best compromise between all the analyzed parameters are those fabricated with either 0.25 or 0.5 mg mL^–1^ of recAb, which were selected for the subsequent studies.

### Application of the 3D-printed immunoextraction device for determination of MC-LR in seawater samples

The 3DF^3^S-recAb ratiometric method for MC-LR was evaluated against microcystin-RR (MC-RR) and nodularin, which are other cyclic cyanotoxins commonly occurring in seawater [30]. At a concentration level of 2.5 µg L^–1^, MC-LR exhibited *ca.* 21.4% signal reduction compared to the unbound, analyte-free, recAb-FITC system, whereas the responses of MC-RR and nodularin were down to *ca.* 3.3% and 2.2% signal decrease, respectively. This demonstrates the selectivity of the F^3^S-based method with no need for tedious column-separation systems [8].

The breakthrough volume of the miniaturized immunoaffinity extraction device was assessed by increasing the sample volume while fixing the mass of MC-LR to 125 ng. Within the range of 50–500 mL, no statistically significant readout differences were detected, with a ratiometric fluorescence intensity decrease, in all cases, of 22.7 ± 1.1% regardless of the sample volume. However, volumes above 500 mL were impracticable because of the limited 3DF^3^S device’s stirring capacity. Increasing the sample volume to 500 mL enabled amelioration of the LOD and LOQ values down to 28 and 94 ng L^–1^, respectively.

As a proof-of-concept applicability, the 3D printed recAb-loaded extraction platform was harnessed to the determination of MC-LR in PBS and synthetic seawater (prepared following the recommendations by Wezel and Likens [31]) at concentration levels equal to or below those endorsed by WHO (viz., 250 and 1000 ng L^–1^) using 500 mL of sample volume. For PBS solutions, the relative recoveries were 108.5 ± 9.6 and 102.9 ± 6.3% at the 250 ng L^–1^, and 1000 ng L^–1^ spike levels, respectively. As to the synthetic seawater, the relative recovery was 91.0 ± 4.5% at 250 ng L^–1^, and 100.4 ± 11.6% at 1000 ng L^–1^. Therefore, high matrix samples with elevated ionic strength do not jeopardize the selective recognition of MC-LR or the extraction capacity of the 3D-printed device. Additionally, the 3DF^3^S-to-3DF^3^S intermediate precision for seawater (< 12%, *n* = 3) was comparable to that of PBS (< 10%, *n* = 3). The devices were used only once because the unbound antibody was modified irreversibly with the fluorescent tag after each extraction. The 3DF^3^S devices incorporating recAb were stable at least for one month whenever stored at – 20 ºC (RSD < 15%, *n* = 6 for 3DF^3^S-to-3DF^3^S precision).

The analytical performance of the proposed 3DF^3^S device for the determination of MC-LR in water samples was compared against that of other published methodologies involving either biosensing or column separation methods (see Table S2 and discussion in Supplementary Information). It should be stressed that most of the reported (bio)analytical systems for the determination of MC-LR in environmental samples were validated for tap, natural, and freshwater systems rather than for high ionic strength matrixes (see Table S2). In fact, one of the main assets of the proposed 3DF^3^S-Ab method is the feasibility of reliable analysis of high matrix samples, such as seawater, without multiplicative matrix interfering effects.

## Conclusions

This manuscript reported on the batch-scale fabrication of 3D-printed multi-functional platforms encompassing selective microscale extraction from large sample volumes (up to 500 mL) under stirring and solid-phase ratiometric fluorescence detection. Additive manufacturing enabled facile adaptation of the microextraction platform to standard instrumentation without hardware modification. The potential of stereolithographic prints for covalent immobilization of biorecognition elements (herein plant-derived antibodies) was fully leveraged. As a proof-of-concept, the 3D-printed modulable device was harnessed to the fluorescence determination of MC-LR at concentrations far below the maximum endorsed levels by WHO, and applied to high matrix samples, such as seawater, without matrix interfering effects. The single-use nature of the recAb-laden device was offset by the ability of sensitive F^3^ optosensing with no need for analyte elution, which is a severe constraint of standard immunosorbents. Another asset of our LFS-based method is the possibility of printing 28 platforms at a time in *ca.* 4 h with an estimated cost of 0.4 €/print, and 10 €/device after recAb immobilization.

### Supplementary Information

Below is the link to the electronic supplementary material.

Supplementary file1 (DOCX 997 KB)

## Data Availability

Data will be available to interested readers upon request.
