# Supplementary material for: Modulable 3D-printed plantibody-laden platform enabling microscale affinity extraction and ratiometric front-face fluorescence detection of microcystin-LR in marine waters
Source: Mikrochim Acta. 2024 Jul 27;191(8):490. doi: 10.1007/s00604-024-06547-2 (PMC11283425; doi:10.1007/s00604-024-06547-2)
Supplement: Supplementary file 1 — Supplementary file1 (DOCX 997 KB) [file 604_2024_6547_MOESM1_ESM.docx]

**Supplementary information**

**Modulable 3D-printed plantibody-laden platform enabling microscale affinity extraction and ratiometric front-face fluorescence detection of microcystin-LR in marine waters**

**Roser Payà-Pou^a^, Julia Aguirre-Camacho^a^, Ernesto Francisco Simó-Alfonso^a^, Dietmar Knopp^b^, Manuel Miró^c*^, Enrique Javier Carrasco-Correa^a*^**

*^a^ CLECEM Group, Department of Analytical Chemistry, University of Valencia, C/ Doctor Moliner, 50, 46100, Burjassot, Valencia, Spain*

*^b^ Technical University Munich, TUM School of Natural Sciences, Department of Chemistry, Chair of Analytical Chemistry and Water Chemistry, Lichtenbergstrasse 4, 85748 Garching, Germany*

*^c^FI-TRACE Group, Department of Chemistry, University of the Balearic Islands, Carretera de Valldemossa, km 7.5, E-07122, Palma de Mallorca, Spain*

*Corresponding authors:*

**Dr. Enrique Javier Carrasco-Correa**
E-mail: [enrique.carrasco@uv.es](mailto:enrique.carrasco@uv.es)
Tel.: +34963544248
Fax: +34963544436

**Prof. Manuel Miró**
E-mail: [manuel.miro@uib](mailto:manuel.miro@uib).es
Tel: +34 971172746
Fax: +34 971173426

**Table of content**

Reagents and materials (p. S3)

Instrumentation (p. S3)

Preparation of plant-derived recombinant antibody against microcystin LR (p. S4)

3D printed surface modification (p. S4)

Fig. S1. Illustration of the reaction scheme for covalent attachment of the plantibody onto the front face surface of the 3D printed device. (p. S5)

Fig. S2. SEM micrographs and percentage of S relative to C on a 3D printed surface (p. S5)

Fig. S3. Logistic sigmoidal ratiometric fluorescence calibration curves obtained with 3DF^3^S devices (p. S6)

Table S1. Analytical parameters of the ratiometric 3DF^3^S methods (p. S7)

Table S2. Analytical performance of alternative bioanalytical methods for the determination of MC-LR in environmental samples (p. S8)

References (p. S11)

**Reagents and materials**

1-ethyl-3-(3-dimethylaminopropyl) carbodiimide (EDC) hydrochloride and N-hydroxysuccinimide (NHS) were purchased from Fisher Scientific (Madrid, Spain). 1,6-hexamethylenediamine (HMD), glutaraldehyde (GA), fluorescein-5-isothiocyanate (FITC) and sodium cyanoborohydride (SNCBH) were obtained from Merck KGaA (Darmstadt, Germany). MC-LR, microcystin-RR and nodularin were purchased from Enzo Life Science (Lausen, Switzerland). All the other reagents employed in this work were of at least technical grade and were supplied by Avantor (Radnor, PA, USA). Deionized water was obtained by the Crystal B30 Adrona deionizer (Riga, Letonia). 3D printed devices were prepared using Clear resin (RS-F2-GPCL-04) purchased from FormLabs (Somerville, MA, USA). A 10 × phosphate buffered saline (PBS) solution containing 1.37 mM NaCl, 27 mM KCl, 80 ·mM Na_2_HPO_4_ and 20 mM KH_2_PO_4_ was prepared in water and adjusted to pH 7.4. Appropriate dilutions were performed based on the experiments required. Synthetic seawater was prepared following the recommendations by Wetzel and Likens [1]. All the solutions containing microcystins were keep at -22 ºC pending use.

**Instrumentation**

The 3D printed F^3^S (3DF^3^S) ratiometric devices were fabricated using a Form 3 3D printer with LFS technology by Formlabs. The post-curing of the printed objects was performed using a UV chamber CL1000 (UVP Inc., Upland, CA, USA) containing 6 UV lamps of 8 W each emitting at 254 nm. The scanning electron microscopy (SEM) characterization of the sensing platform was conducted with a SCIOS2 FIB-SEM (ThermoFisher Scientific, MA, USA). Fluorescence optosensing of the recombinant monoclonal plantibody (recAb)-containing solid materials was performed by the spectrofluorometer MOD FP-6200 (Jasco, Madrid, Spain) equipped with a Xe lamp. The bandwidths of excitation and emission were configurated at 5 nm and the gain was set at medium level (approx. ×10).

**Preparation of plant-derived recombinant antibody against microcystin LR**

The procedure for the synthesis, isolation, purification, and molecular characterization of the recAb against MC-LR was described elsewhere [2]. It should be noted that the recombinant plantibody was obtained from *Nicotiana benthamiana* and relied upon the mouse monoclonal antibody MC10E7 [3]. Leaves of *Nicotiana benthamiana* revealed to be most suitable for the rapid production of large quantities of the full-size recAb. The best reproducible yields after purification reached 329 mg of purified antibody per kg fresh weight of leaf material.

**3D printed surface modification**

The covalent surface functionalization of the 3DF^3^S device was merely performed on the front face of the main unit following the optimized multi-step procedure described elsewhere [4] and schematized in Fig. 2. Briefly, the generation of carboxylic acid moieties on the acrylate print surface was enabled by reaction with a 2 mol/L aqueous NaOH solution at 60 °C for 30 min [4,5]. Then, the prints were cleaned with 0.1 mol/L HCl and water and left to air dry. A further esterification protocol was conducted using an aqueous solution containing 0.2 mM EDC and 0.3 mM NHS at 60 °C for 30 min, followed by reaction for 1 h at 60 °C with 0.52 mM aqueous HMD. After each step, the functionalized wall was cleaned with water, and then dried with a stream of N_2_. Afterwards, 100 µL of a 50% glutaraldehyde solution was added onto the 3D printed front face for 12 hours at room temperature. The device was again cleaned with water and dried at room temperature. Subsequently, the recAb was anchored to the front face surface using 100 µL of a 0.25 or 0.5 mg·mL^–1^ antibody solution in 10 mM PBS for 12 h. Finally, after cleaning, the recAb-immobilized print was reacted with 0.2 M SCNBH in 10 mM PBS for 2 h at 4 ºC, which was aimed at reducing the double bonds. Finally, the 3D printed immunosorbent devices were dried with a stream of N_2_ and stored at -20 ºC pending use.

**
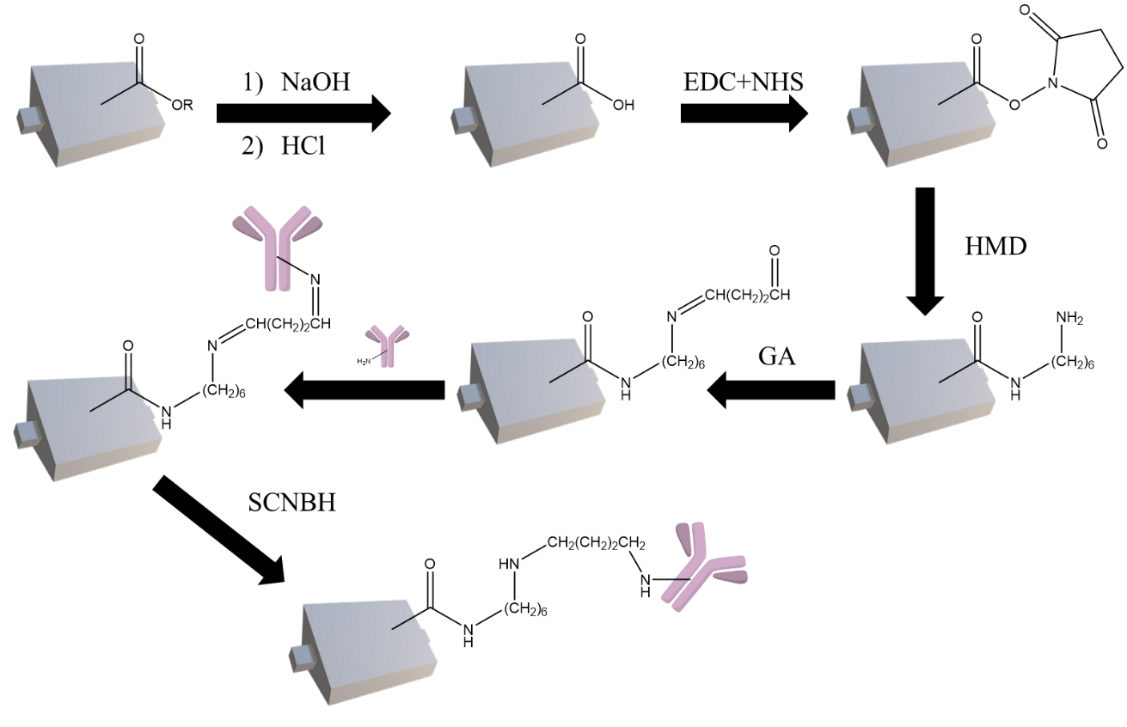
**

**Fig. S1.** Illustration of the reaction scheme for covalent attachment of the plantibody onto the front face surface of the 3D printed device.


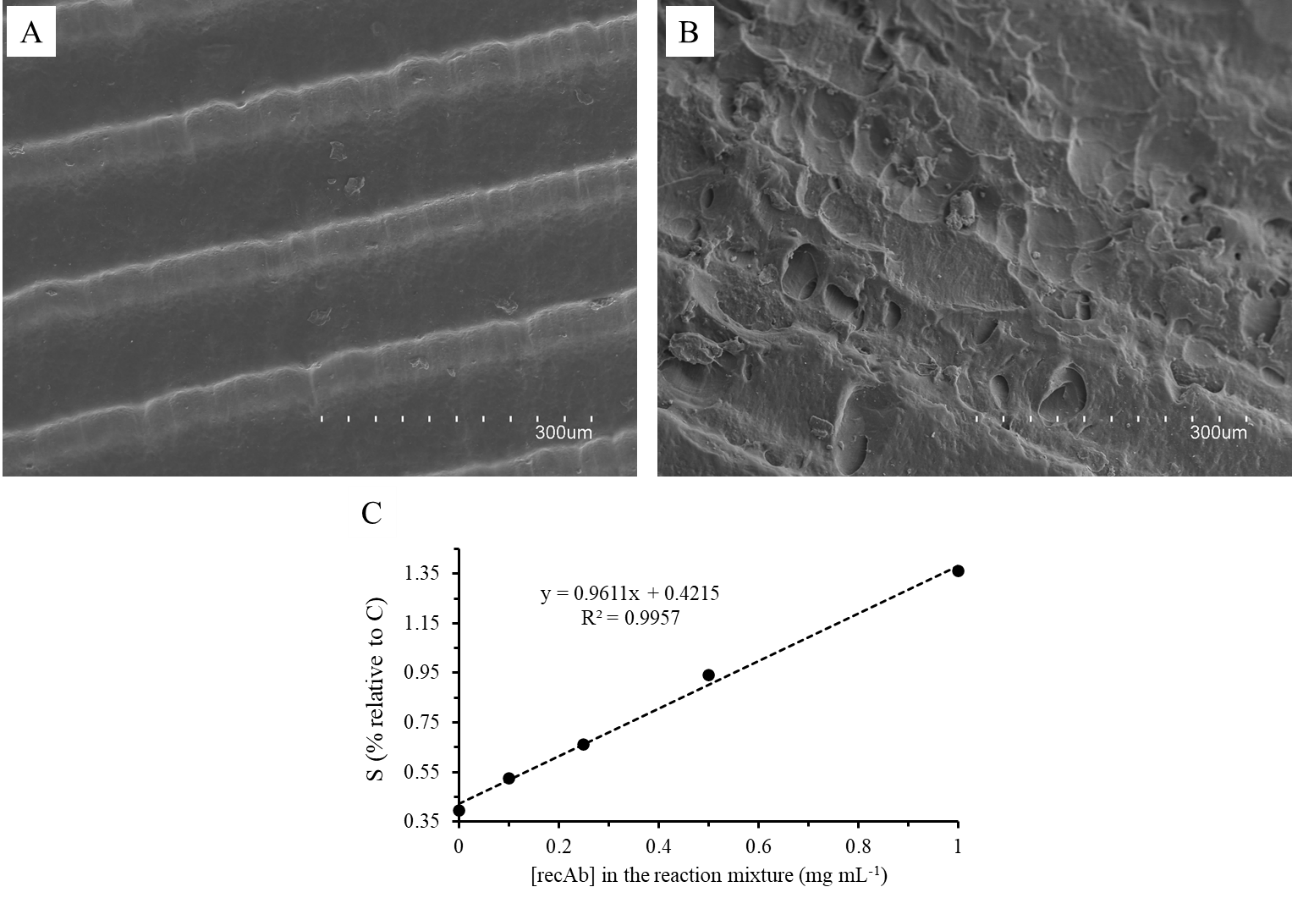


**Fig. S2.** SEM micrographs at 180× of the pristine (A) and the rec-Ab modified (B) 3DF^3^S devices, and the percentage of S relative to C on a 3D printed surface (detection window of 1 µm × 1 µm), which is directly proportional to the amount of rec-Ab in the reaction solution (C).

**Fig. S3.** Logistic sigmoidal ratiometric fluorescence calibration curves obtained with 3DF^3^S devices fabricated using different amounts of recAb: 0.1 (A), 0.25 (B), 0.5 (C) and 1 mg·mL^–1^ (D) with a standard volume of 50 mL. Errors bars are given as standard deviation values (n = 3).

**Table S1.** Analytical parameters of the ratiometric 3DF^3^S methods using increasing concentrations of recAb in the reaction mixture.

| **[recAb]**  **(mg·mL^-1^)** | **LOD**  **(µg·L^-1^)** | **LOQ**  **(µg·L^-1^)** | **RSD**  **(%)** | **Linear range**  **(µg·L^-1^)** | **Linear equation** |
| --- | --- | --- | --- | --- | --- |
| 0.1 | 0.17 | 0.56 | 3.2-21.6 | 0.6-1.00 | $\frac{I_{FC}}{I_{FC0}}=0.408-0.57\cdot Log\left[ MC-LR \right](\mu g L^{-1})$  R^2^ = 0.997 |
| 0.25 | 0.28 | 0.94 | 3.1-7.8 | 1.0-2.5 | $\frac{I_{FC}}{I_{FC0}}=0.697-0.34\cdot Log\left[ MC-LR \right](\mu g L^{-1})$  R^2^ = 0.999 |
| 0.5 | 0.48 | 1.59 | 3.0-7.6 | 1.6-10.0 | $\frac{I_{FC}}{I_{FC0}}=1.21-1.02\cdot Log\left[ MC-LR \right](\mu g L^{-1})$  R^2^ = 0.991 |
| 1.0 | 1.38 | 4.6 | 1.4-7.4 | 4.6-15.0 | $\frac{I_{FC}}{I_{FC0}}=1.42-1.14\cdot Log\left[ MC-LR \right](\mu g L^{-1})$  R^2^ = 0.943 |

**Table S2.** Analytical performance of alternative bioanalytical methods for the determination of MC-LR in environmental samples

| **Method** | **LOD**  **(ng·L^-1^)** | **RSD**  **(%)** | **Recoveries ([MC-LR], ng·L^-1^)**  **(%)** | **Matrixes** | **Reference** |
| --- | --- | --- | --- | --- | --- |
| ELISA kit | 34 | 7.8-9 | 82.4-121.2 (100-2,500) | Sanitary waters | [6] |
| Protein phosphatase inhibition | 10 | - | 84-100 (20,000-100,000) | Natural bloom samples | [7] |
| ImmunoSPE-HPLC-UV | 17^1^ | 9.7-9.9 | 104.5-106.5 (0.16-0.48 mg algae) | Algae extract | [8] |
| ImmunoSPE-HPCE-UV | 13^1^ | 3.5 |  | Algae extract |  |
| UPLC-MS/MS | 100 | <5 | 88-110 (500-70,000) | Lake waters | [9] |
| SPE-HPLC-MS/MS | 2.1 | 7.5-10.6 | 123.1-130.7 (7-150) | Reservoir water | [10] |
| IL-DLLME-HPLC-MS | 3 | 10.7-10.9 | 45-109.7 (1,000-50,000) | Tap and river water | [11] |
| DBCO-aptamer modified MOF SPME-HPLC-MS | 3 | 2.5-14.3 | 94.4-98.9 (8-1,000) | Tap, pond and river waters | [12] |
| Electrochemical immunosensor | 5 | 0.9-3.3 | 98.3-102.1 (10-20,000) | Tap, drinking and lake waters | [13] |
| Chemiresistive aptasensor | 0.18 | 4.08 – 7.82 | 98.42 – 106.98 (1-10,000) | Tap, river, lake and reservoir waters | [14] |
| Electrochemical sensor based on AuNPs@MWCNTs/GQDs | 2.7 | 3.18 – 6.3 | 91.4 – 116.7 (80-20,000) | Tap water | [15] |
| Fluorescence aptasensor | 2 | - | 94-112 (10-50,000) | Tap and lake waters | [16] |
| Fluorescence magnetic-based immunosensor | 0.012 | 6.6-11.5 | 88.5-108 (250-25,000) | Drinking water | [17] |
| Electrochemical immunosensor | 0.11 | 2.3-11.9 | 86-103 (500-2,000) | Drinking water | [18] |
| 3DF^3^S extractor-immunosensing platform | 28 | 4.5-11.6 | 91.0-108.5 (250-1,000) | Seawater | This method |

Acronyms: LOD: Limit of detection; LOQ: Limit of quantification; ELISA: Enzyme-Linked ImmunoSorbent Assay, SPE: Solid-Phase Extraction, HPLC: High-Performance Liquid Chromatography, UV: Ultraviolet, HPCE: High-Performance Capillary Electrophoresis, UPLC: Ultra Performance Liquid Chromatography, MS: Mass Spectrometry , IL-DLLME: Ionic Liquids in Dispersive Liquid-Liquid Microextraction, DBCO: dibenzocyclooctyne, MOF: Metal-Organic Framework, SPME: Solid Phase MicroExtraction, AuNPS: Gold nanoparticles, MWCNT: Multi-Walled Carbon NanoTubes, GQD: Graphene Quantum Dots

^1^ ng MC-LR·g^-1^ algae extract

As it can be seen in Table S2, the LOD of the 3D F3S-recAb method equates those of ELISA and immunoSPE counterparts [6-8] but it is one order of magnitude better than that achieved with bulk UPLC-MS/MS equipment [9]. It is also just slightly higher than that obtained using tedious and less sustainable methodologies combining sorptive or liquid-phase extraction with HPLC-MS [10-12]. Other sophisticated biosensing schemes showed around one order of magnitude lower LODs [13-16]. In some other cases [17,18], spike concentrations in drinking waters were akin to our proposed method despite the reported LODs were significantly inferior. In addition, it should be stressed that most of the reported (bio)analytical systems for determination of MC-LR in environmental samples are validated for tap, natural and freshwater systems rather than for high ionic strength matrixes (see Table S2). In fact, our 3DF^3^S-Ab device enables reliable analysis of high matrix samples, such as seawater, without multiplicative matrix interfering effects.

**References**

[1] Wetzel RG, Likens GE (2000) Limnological Analyses, Springer, New York, NY. <https://doi.org/10.1007/978-1-4757-3250-4>.

[2] Melnik S, Neumann AC, Karongo R, Dirndorfer S, Stübler M, Ibl V, Niessner R, Knopp D, Stoger E (2018) Cloning and plant-based production of antibody MC10E7 for a lateral flow immunoassay to detect [4-arginine]microcystin in freshwater, Plant Biotechnol. J. 16:27–38. https://doi.org/10.1111/pbi.12746

[3] Zeck A, Eikenberg A, Weller MG, Niessner R (2001) Highly sensitive immunoassay based on a monoclonal antibody specific for [4-arginine]microcystins, Anal. Chim. Acta. 441:1–13. https://doi.org/10.1016/S0003-2670(01)01092-3.

[4] Carrasco-Correa EJ, Herrero-Martínez JM, Simó-Alfonso EF, Knopp D, Miró M (2022) 3D printed spinning cup-shaped device for immunoaffinity solid-phase extraction of diclofenac in wastewaters, Microchim. Acta. 189:173. https://doi.org/10.1007/s00604-022-05267-9.

[5] Carrasco-Correa EJ, Cocovi-Solberg DJ, Herrero-Martínez JM, Simó-Alfonso EF, Miró M (2020) 3D printed fluidic platform with in-situ covalently immobilized polymer monolithic column for automatic solid-phase extraction, Anal. Chim. Acta. 1111:40–48. https://doi.org/10.1016/j.aca.2020.03.033.

[6] Smienk HGF, Sevilla E, Peleato M, Razquin P, Mata L (2007) Kit validation for Microcystins detection in waters (Spanish), Alimentaria, 104-111.

[7] Ward CJ, Beattie KA, Lee EYC, Codd GA (1997) Colorimetric protein phosphatase inhibition assay of laboratory strains and natural blooms of cyanobacteria: comparisons with high-performance liquid chromatographic analysis for microcystins, FEMS Microbiol. Lett. 153:465–473. https://doi.org/10.1111/j.1574-6968.1997.tb12611.x.

[8] Aguete EC, Gago-Martı́nez A, Leão JM, Rodrı́guez-Vázquez JA, Menàrd C, Lawrence JF (2003) HPLC and HPCE analysis of microcystins RR, LR and YR present in cyanobacteria and water by using immunoaffinity extraction, Talanta. 59:697–705. https://doi.org/10.1016/S0039-9140(02)00610-0.

[9] Oehrle SA, Southwell B, Westrick J (2010) Detection of various freshwater cyanobacterial toxins using ultra-performance liquid chromatography tandem mass spectrometry, Toxicon. 55:965–972. https://doi.org/10.1016/j.toxicon.2009.10.001.

[10] Aparicio-Muriana MM, Carmona-Molero R, Lara FJ, García-Campaña AM, Del Olmo-Iruela M (2022) Multiclass cyanotoxin analysis in reservoir waters: Tandem solid-phase extraction followed by zwitterionic hydrophilic interaction liquid chromatography-mass spectrometry, Talanta. 237:122929. <https://doi.org/10.1016/j.talanta.2021.122929>.

[11] Yu H, Clark KD, Anderson JL (2015) Rapid and sensitive analysis of microcystins using ionic liquid-based in situ dispersive liquid–liquid microextraction, J. Chromatogr. A. 1406:10–18. https://doi.org/10.1016/j.chroma.2015.05.075.

[12] Xu Z, Zhang Z, She Z, Lin C, Lin X, Xie Z (2022) Aptamer-functionalized metal-organic framework-coated nanofibers with multi-affinity sites for highly sensitive, selective recognition of ultra-trace microcystin-LR, Talanta. 236:122880. https://doi.org/10.1016/j.talanta.2021.122880.

[13] Zhang Y, Chen M, Li H, Yan F, Pang P, Wang H, Wu Z, Yang W (2017) A molybdenum disulfide/gold nanorod composite-based electrochemical immunosensor for sensitive and quantitative detection of microcystin-LR in environmental samples, Sens. Actuators B 244:606–615. https://doi.org/10.1016/j.snb.2017.01.030.

[14] Liu J, Tan F, Xing Y, Zhang Q, Zhao Z, Wang X, Wang Y, Zhao H (2023) Label-Free Chemiresistive Sensors Based on Self-Assembled Ti3C2Tx MXene Films for Monitoring of Microcystin-LR in Water Samples, Environ. Sci. Technol. 57:15432–15442. https://doi.org/10.1021/acs.est.3c05791.

[15] Zhao R, Li J, Wu C, Cai J, Li S, Li A, Zhong L (2023) Reaction mechanism and detecting properties of a novel molecularly imprinted electrochemical sensor for microcystin based on three-dimensional AuNPs@MWCNTs/GQDs, Water Sci. Technol. 88:572–585. https://doi.org/10.2166/wst.2023.238.

[16] Lv J, Zhao S, Wu S, Wang Z (2017) Upconversion nanoparticles grafted molybdenum disulfide nanosheets platform for microcystin-LR sensing, Biosens. Bioelectron. 90:203–209. <https://doi.org/10.1016/j.bios.2016.09.110>.

[17] Guan T, He J, Liu D, Liang Z, Shu B, Chen Y, Liu Y, Shen X, Li X, Sun Y, Lei H (2020) Open Surface Droplet Microfluidic Magnetosensor for Microcystin-LR Monitoring in Reservoir, Anal. Chem. 92:3409–3416. https://doi.org/10.1021/acs.analchem.9b05516.

[18] Guan T, Huang W, Xu N, Xu Z, Jiang L, Li M, Wei X, Liu Y, Shen X, Li X, Yi C, Lei H (2019) Point-of-need detection of microcystin-LR using a smartphone-controlled electrochemical analyzer, Sens. Actuat. B. 294:132-140, https://doi.org/10.1016/j.snb.2019.05.028.
